# Recent Applications of Photothermal Conversion in Organic Synthesis

**DOI:** 10.1021/acscentsci.4c00545

**Published:** 2024-08-05

**Authors:** Megan E. Matter, Clotilde Tagnon, Erin E. Stache

**Affiliations:** † Department of Chemistry, Princeton University, Princeton, New Jersey 08544, United States

## Abstract

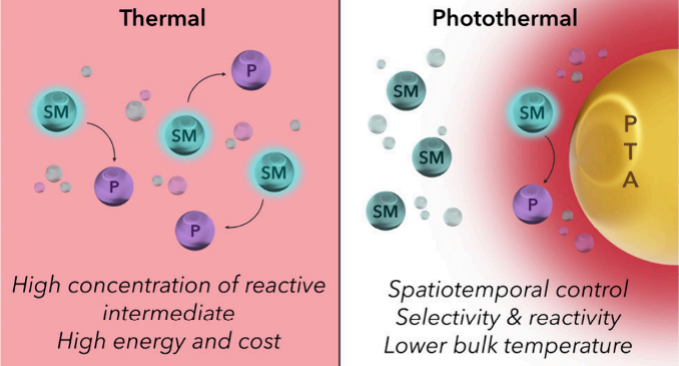

Photothermal conversion is a novel heating method that has emerged in recent years, wherein certain species can convert light to heat with great efficiency. These photothermal agents have shown immense promise for generating nanoscale thermal gradients under mild, visible light irradiation, providing a pathway for combining photochemistry with thermally driven reactivity. While this novel heating mechanism has been leveraged to great effect for applications such as photothermal therapeutics and steam water purification, it has seen limited use in organic synthesis. This outlook explores instances wherein the photothermal effect was used directly or as a synergistic component to drive organic reactions and postulates how it may be used moving forward.

## Abstract 2

Photothermal conversion has emerged as a method for applying high heat under irradiation. This outlook explores how it has been used in synthesis and its potential use in future applications.

The discovery and development of new methodologies for enabling novel chemical reactivity are fundamental components of organic synthesis. The efficient production of commodity chemicals, pharmaceuticals, and agrochemicals and the often-difficult transformations that empower their production are essential to everyday life.^1−3^ Chemists have pursued increasingly more atom-economical, robust, and selective chemical transformations toward these aims. Many approaches use catalyst development, where species such as transition metals, metal–organic frameworks, enzymes, etc. lower the activation energy barrier for a given transformation.^4−6^ In doing so, catalysts can promote reactions that otherwise would not occur or would occur on a time scale so prohibitively long that their use becomes impractical. Although there have been significant advances in this area, many reactions still require additional energetic stimulus, often supplied as heat. Adding heat does not help lower the energetic barrier for a reaction; instead, it assists molecules in climbing that barrier.

The effect temperature has on the relative rates of a reaction is well established, with quantitative descriptions first reported by Van’t Hoff and Arrhenius as early as the late 1800s.^7,8^ Their critical insight was how temperature changes affected the equilibrium population of reactive species. Thermal energy remains an essential component of both catalytic and uncatalyzed reactions, with many of the well-known and industrially applicable reactions (olefin hydrogenation, pyrolysis/cracking, etc.) relying on significant thermal input alongside catalyst incorporation.^9−12^ Because of the ease with which technology allows scientists to apply a wide range of temperatures with a high degree of consistency and accuracy, little attention has been paid to unique forms of generating/applying thermal energy in organic synthesis. Additionally, the high concentration of reactive intermediates produced by bulk heating can lead to deleterious side reactivity, discouraging its implementation. The ability to apply localized heating has not been broadly explored in synthesis.

Innovations within the nanomaterials and photochemistry fields have led to the discovery and implementation of materials that exhibit the photothermal effect—a novel heating method with the potential to overcome these challenges.^13−16^ The photothermal effect arises from unique energetic decay pathways which enable the conversion of photonic energy into heat. This diverse array of species can be sorted into three general types: plasmonic metallic nanostructures, nonplasmonic semiconductors, and organic materials such as conjugated organic dyes and carbon-based nanomaterials (Figure 1).

**Figure 1:**
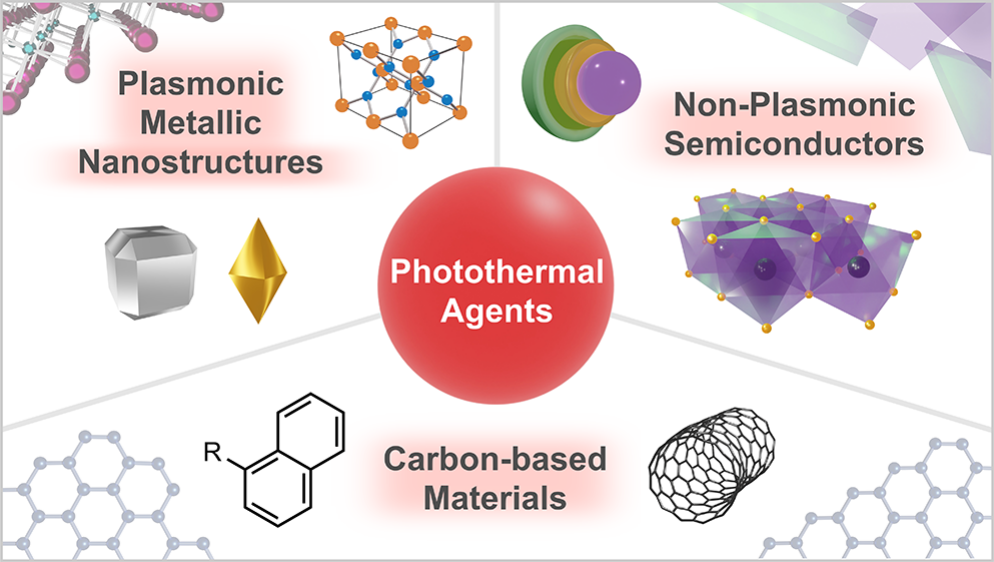
An overview of the major types of photothermal agents.

While these materials and their mechanisms for undergoing photothermal conversion are distinct, the overall effect is the same—the conversion of light into heat. Photothermal heating is unique compared to traditional bulk heating because of its photomediation and thermal gradients.^17,18^ The temperature near the surface of a photothermal agent dramatically exceeds that of the bulk solution, and the radiant heat decays exponentially upon diffusion from the particle. In contrast to bulk heating, where a rate constant is uniform across the reaction media, reaction rates are variable in photothermal heating, fastest near the nanoparticle and decreasing with distance—the substrate distal from the surface is nonreactive. While this may seem counterintuitive to the enhanced rates observed in many photothermally driven reactions, the nanoscale heat simultaneously aids diffusion. Therefore, the substrate interacts with the particle surface efficiently, improving overall reactivity. The photothermal effect has so far seen widespread use in biomedical and inorganic materials applications.^19−21^ The ability to generate highly reactive species while limiting total concentration and inhibiting promiscuous side reactivity presents a promising avenue for promoting challenging, high-activation-energy barrier transformations. This Outlook aims to highlight photothermal conversion used in organic synthesis and identify its potential as a platform for enabling challenging reactivity.

## 1 Photothermal Conversion and Photothermal Agents

Photothermal conversion can simply refer to any species that, when exposed to light, converts that energy to heat through some mechanism. The process by which the material undergoes conversion is highly structure-dependent. In this section of the outlook, we will cover the three primary classes of photothermal agents, as their structure and distinct properties help inform the kind of reactivity they can promote. Plasmonic metallic nanostructures are typically composed of precious metals, such as Au, Ag, Pt, etc. These species exhibit localized surface plasmon resonance (LSPR) when exposed to structure-specific wavelengths of light.^22−25^ The mechanism by which these species undergo photothermal conversion is relatively complicated and remains an ongoing subject of debate. Several reviews and papers extensively detail the proposed mechanisms and experimental evidence, and in deference to those reports, we will provide a simplified overview of what others have previously articulated.^20−23^

**Figure 2:**
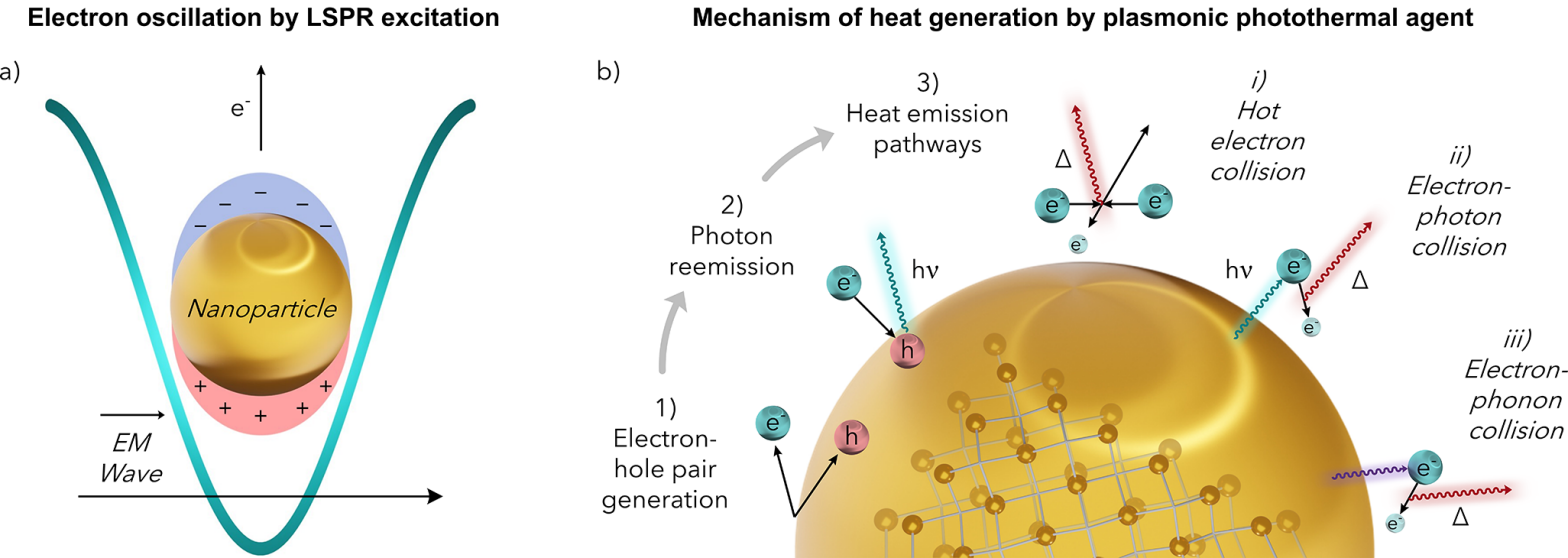
a) Pictorial representation of localized surface plasmon resonance, wherein electron clouds oscillate opposite the incoming electromagnetic wave. b) Sequence of events that leads to heat emission in plasmonic materials.

In a plasmonic nanostructure, the absorption of a photon identical in energy to its LSPR frequency induces the excitation of free electrons oscillating within the nanostructure, bringing them to the surface of the particle (Figure 2a). The surface electronic oscillation produces a nonequilibrium among the electrons, and several processes begin to occur rapidly to dissipate the excess energy.^22−25^ Photons are re-emitted, electron–hole pairs are generated via Landau damping, and hot electron collisions occur. The latter two processes produce a Fermi–Dirac distribution of electrons, contributing to an eventual thermal emission from the plasmon’s surface. This intricate chain of events allows plasmonic materials to act as photothermal conversion agents (Figure 2b). Photothermal conversion is a tertiary process within the plasmonic structure, functioning only to reallocate energetic excess within the system. Because of plasmonic nanostructures’ ability to engage in energy-transfer events and excite nearby molecules, photothermal conversion is often seen as synergistic and not the primary driving force for reactivity.

Plasmonic materials, while incredibly useful and capable of highly unique reactivity patterns, rely on more complex nanoparticle architectures and typically require a single wavelength to induce productive LSPR events.^26−28^ Plasmonic materials are frequently excited with laser irradiation, limiting user-friendliness. Several recent reports have attempted to rectify the high degree of wavelength specificity by utilizing multiple shapes and sizes of the nanoparticle in a given reaction mixture, broadening the overall absorption profile. Some plasmonic materials have been rendered amenable to broad-spectrum LEDs and even sunlight irradiation using these methods. Heterogeneous scaffolds for plasmonic species and protective coatings have also been used for catalyst recovery and longevity, reducing long-term costs. Despite these advances, the overall cost of material and difficulty of synthesis remain challenges for the widespread implementation of plasmonic materials.

Metallic semiconductors can also act as photothermal agents. However, they are infrequently used in organic synthesis due to decreased photothermal efficiency compared to that of plasmonic and carbon-based photothermal agents. They are more frequently used for solar water purification, photothermal therapeutics, and photoacoustic applications.^14,29,30^

**Figure 3:**
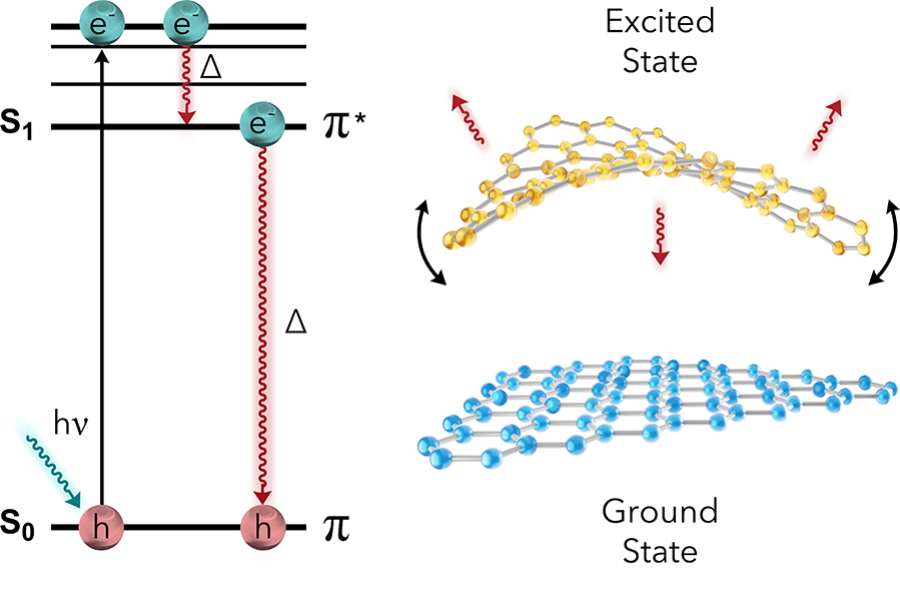
Excitation and heat generation pathways for carbon-based photothermal materials.

While the prior photothermal agents mentioned rely on the high availability of free electrons in metals, a wide variety of carbon-based photothermal agents generate heat by entirely different mechanisms.^31−35^ Two significant categories exist within this subclass: carbon nanoparticles and molecular photothermal agents. Carbon nanoparticles can range from 1-D to 3-D nanostructures composed of organized carbon atoms with size-dependent properties. The structural motif unifying these diverse materials is an abundance of loosely held π electrons. Unlike metallic nanostructures, most carbon-based photothermal agents are broadband absorbers. The π electrons are promoted to energetically similar π* orbitals when irradiated. The minor energy differences between these π and π* orbitals result in rapid internal conversion and intersystem crossing events (Figure 3). This conversion is manifested in bond vibrations and rotations, resulting in localized areas of intense nanoscale heat.^29−33^ Molecular photothermal agents, such as heptamethylene blue and indocyanine green, use this same pathway for undergoing photothermal conversion. These species typically consist of conjugated C–C bonds, commonly arranged in a flexible macrocyclic structure, allowing for productive photothermal conversion. They are frequently used in photothermal therapeutics but remain largely absent from synthetic methodologies.^36,37^

In contrast to their metallic counterparts, the bulk of carbon-based photothermal agents cannot undergo alternative energy-transfer pathways. This limitation makes them an ideal mediator for thermally driven reactions, as they are otherwise often chemically inert. Additionally, many carbon-based photothermal agents are inexpensive and avoid specialty synthesis methods. Materials such as carbon nanotubes or graphene sheets can rely on laborious preparation methods. However, they are derived from inexpensive carbonaceous materials. Additionally, many carbon-based photothermal agents come from industrial byproducts (i.e., carbon black), making them affordable and widely available. Carbon-based photothermal agents, however, have seen limited use, and the extent of their ability to generate productive heat is demonstrated in only a few reports.

## 2 Hydrogenation and Oxidation

While photothermal conversion has seen limited use as the sole force for driving organic reactions, it has gained attention for its synergistic function within a catalytic system. Numerous transformations proceed at high temperatures despite catalyst incorporation. However, high-temperature bulk reactors can be a safety hazard, are energetically costly, and often result in unwanted side reactivity. Incorporating a photothermal agent, which produces localized heat and minimal bulk temperature increase, offers immense advantages in terms of efficiency, catalyst performance, and operational hazards. Several reports have realized these advantages, using this technique to achieve a diverse array of challenging hydrogenation and oxidation reactions.

**Figure 4:**
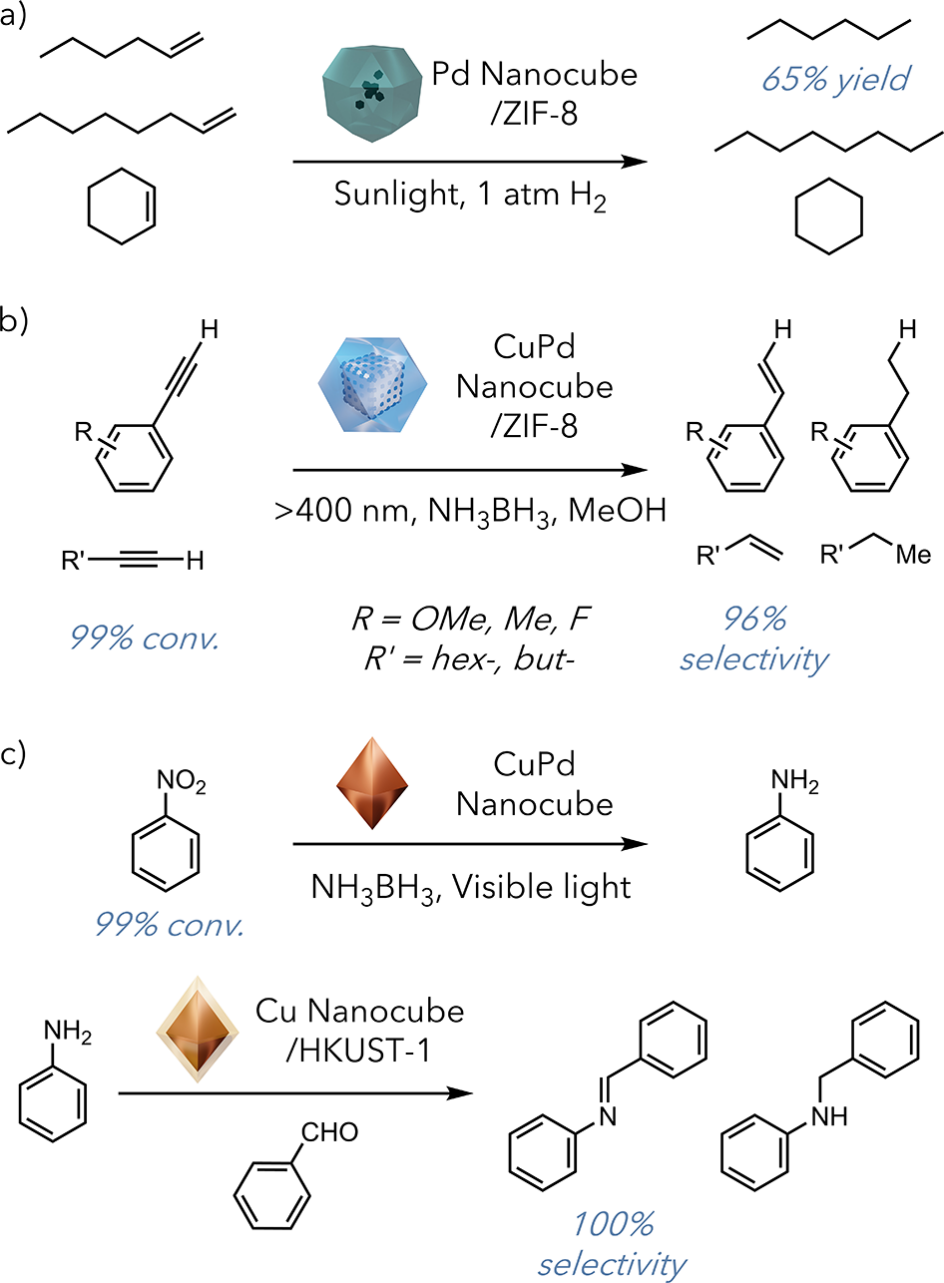
a) Selective hydrogenation of short-chain alkenes. b) Selective hydrogenation of alkynes. c) Tandem nitrobenzene reduction/reductive amination of benzaldehyde.

The Jiang laboratory has made notable contributions in this area, with a preliminary report detailing Pd nanocubes embedded in the ZIF-8 MOF to perform the hydrogenation of terminal alkenes (Figure 4a).^38^ The Pd nanocubes functioned as both a photothermal agent and a reductive catalyst. They demonstrated the positive influence of photothermal heating on the reaction, wherein irradiative room-temperature conditions produced hydrogenation efficiencies identical to those used at elevated temperatures (∼50 °C). While this is a modest difference in temperature, it remains a seminal example of the synergistic relationship between photothermal conversion and catalytic processes. Many hydrogenation reactions use a Pd catalyst, so incorporating a photothermally active Pd species enables a photocontrolled protocol with operational advantages.

Jiang elaborated on this initial work by developing a bimetallic nanoparticle that could partially hydrogenate terminal alkynes (Figure 4b).^39^ Overhydrogenation is a recurrent challenge in organic synthesis due to the high reactivity of the terminal alkene, which results from the initial hydrogenation of the alkyne. They found that embedding CuPd nanoparticles into the ZIF-8 MOF provided quantitative conversion and excellent selectivity (96%) for converting phenylacetylene to styrene in just 3 minutes. Photothermal heating is uniquely valuable in microporous structures such as MOFs as it enhances substrate diffusion and increases collision rates. Enhanced diffusion improved the reaction efficiency, enabling the implementation of a nongaseous hydrogen source, ammonia borane, providing additional advantages. This strategy was also applied in a later report with a copper nanostructure to induce tandem nitroarene reduction and subsequent reductive amination of benzaldehyde to induce selective imine formation (Figure 4c).^40^ These initial examples demonstrated some of the advantages of using photothermal materials. The inherent limitation to these approaches lies in the use of a specialty nanoparticle which must be presynthesized, and additionally requires the synthesis and embedding within an MOF scaffold. This presents a challenge for future industrial application and long-term applicability, as not only are these processes highly technical but they can also be subject to difficulties in scaling and cost.

**Figure 5:**
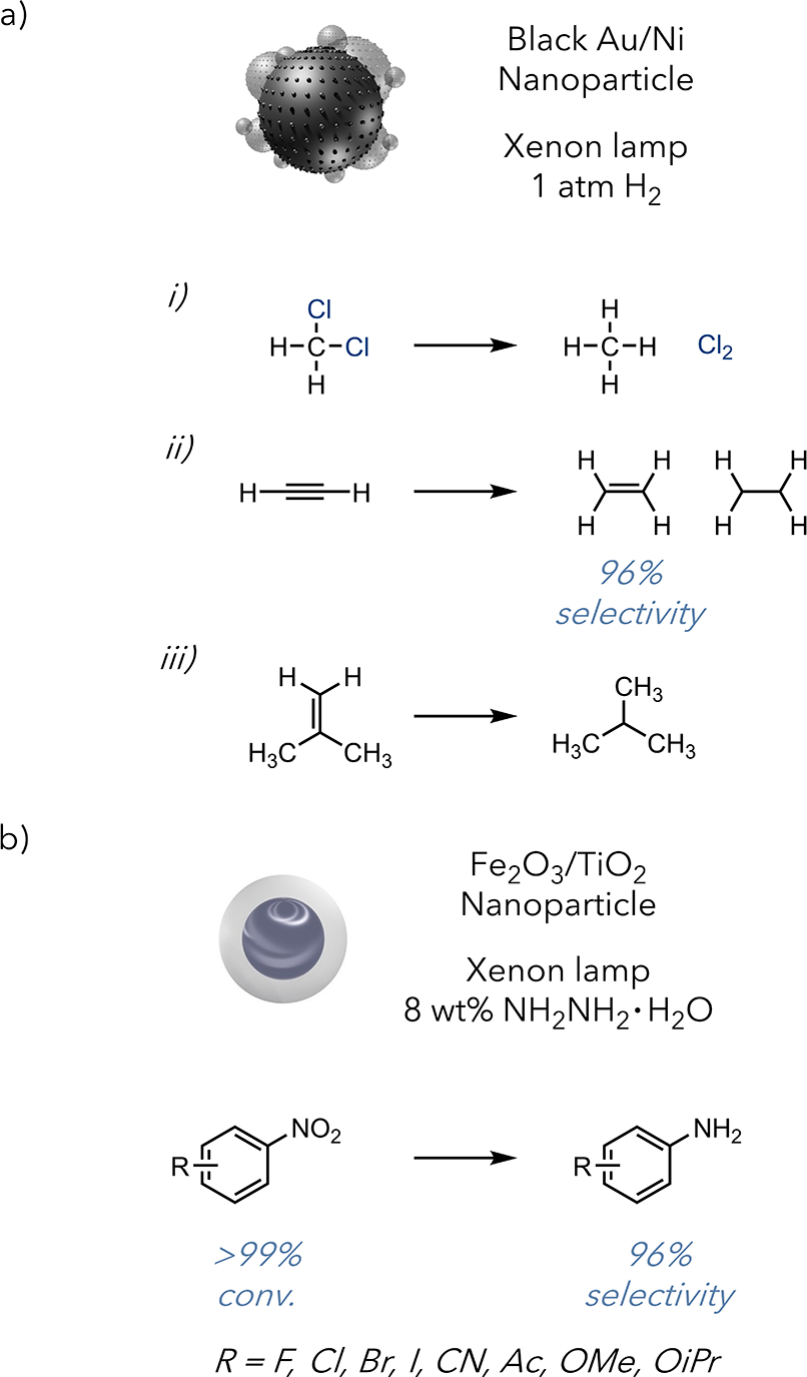
a) Black Au/Ni nanoparticle irradiated by a xenon lamp in 1 atm H_2_ to facilitate (i) hydrodechlorination of dichloromethane, (ii) selective hydrogenation of acetylene to ethene, and (iii) hydrogenation of propene. b) Selective hydrogenation of nitroarenes.

The Polshettiwar group recently reported a non-MOF-based approach to utilizing plasmonic nanostructures for selective hydrogenation. They showed a novel black Au–Ni nanoparticle that performed the selective hydrogenation of acetylene and isobutylene (Figure 5a).^41^ A notable feature of this work was their extension of this approach to the hydrodechlorination of dichloromethane—reactivity which has remained elusive for solely thermal or plasmonic approaches. They also experimentally determined the relative photothermal and plasmonic contributions, finding that 245 °C external heating was required to achieve similar methane production rates when the reaction was run in the dark. While there are a number of researchers working to deconvolute plasmonic versus photothermal effects, this report, along with several others, demonstrates the unique reactivity accessible in these regimes.^42,43^ The combined synergistic plasmonic and photothermal effects enable reactivity which was previously inaccessible. This report demonstrates the potential for photothermal conversion to promote challenging reactivity. These experiments suggest that the local temperature of the nanoparticle exceeds 245 °C under mild visible light irradiation while exhibiting a much lower bulk temperature. This process is performed industrially at temperatures exceeding 400 °C in conjunction with a heterogeneous catalyst. These processes usually achieve 70–80% selectivity for methane. In contrast, Polshettiwar’s method relies solely on light irradiation of the catalyst and achieves 100% methane selectivity using a specialized catalyst.

A recent report from the Zhao and Xiong groups detailed the use of a low-cost and high-performing Fe_3_O_4_–TiO_2_ catalyst, which was able to successfully hydrogenate a diverse array of nitroarenes to afford the corresponding amine (Figure 5b).^44^ Iron and titanium-based photothermal agents offer a more inexpensive and accessible catalyst, while addressing challenging reactivity. Notably, they found that interactions between Fe_3_O_4_ and TiO_2_ produced an enhanced photothermal effect, which they attribute to strong metal interface–support interactions, enabling strong absorption and efficient nonradiative emission. They were successfully able to scale the reaction as well, demonstrating that photothermally promoted and industrially relevant processes, such as the hydrogenation of a diverse array of nitroarenes, can function under industrially relevant conditions.

**Figure 6:**
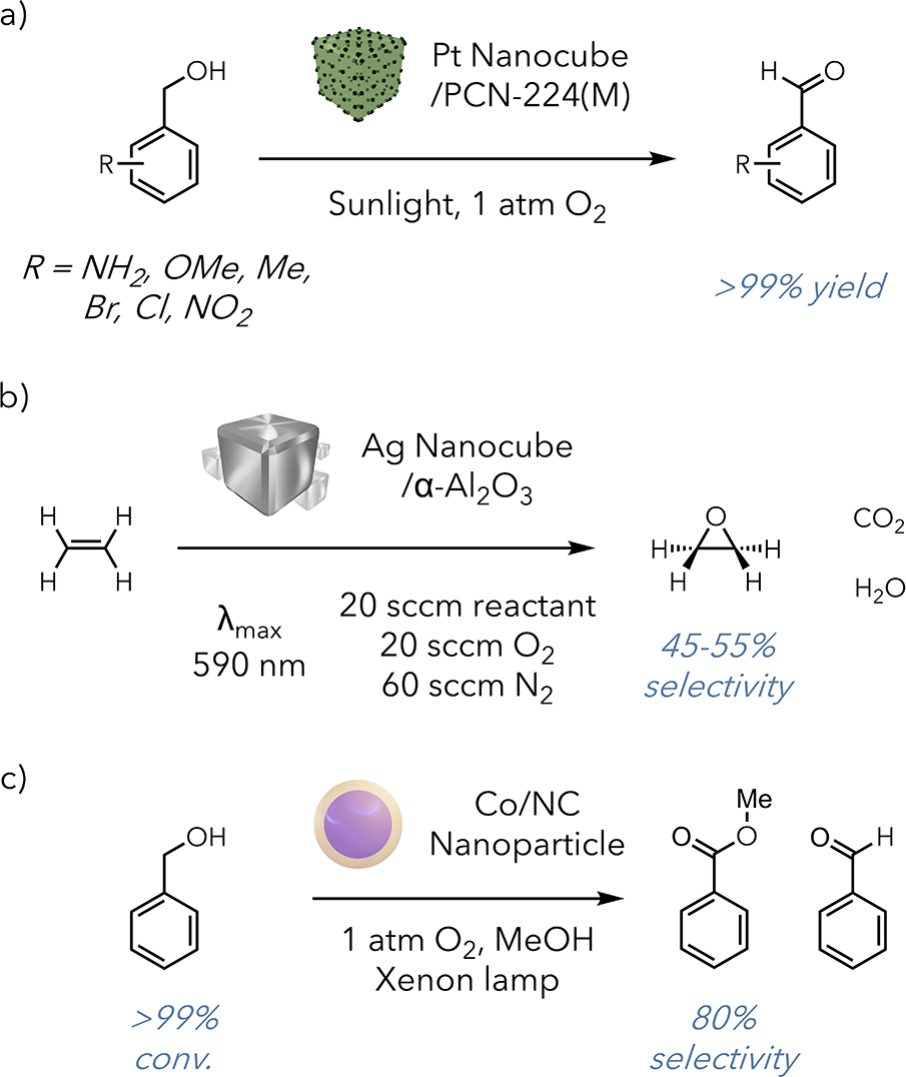
a) Selective oxidation of benzylic alcohols to aldehydes. b) Oxidation of ethylene to ethylene oxide. c) Selective oxidation of benzyl alcohol to methyl benzoate.

Catalytic oxidation reactions (ethylene epoxidation, CO oxidation, etc.) represent another industrially relevant but challenging transformation class. The Jiang group applied their plasmonic MOF approach to selectively oxidize aromatic alcohols to aldehydes (Figure 6a).^45^ They used a different plasmonic material, Pt nanocrystals, and a different MOF, PCN-224, for this transformation. They observed similar reaction efficiency and selectivity increases in their hydrogenation protocol. The general applicability of this approach has also been shown in several other reports that have successfully used photothermal MOFs. As previously mentioned, photothermal heating proves uniquely valuable to porous, heterogeneous catalysts such as MOFs, which can suffer from poor interaction between substrate and catalyst. Under bulk heating, the yields were severely reduced in this system compared to in the photocontrolled system, further supporting this idea.

Photothermal agents have additionally demonstrated value in promoting catalytic oxidation reactions outside a MOF scaffold. In 2011, the Linic group successfully performed several challenging oxidation reactions, showing a 3–4-fold rate enhancement compared to the rate of traditional methods (Figure 6b).^46^ They could also use standard LEDs instead of laser light. There is vast potential for implementing photothermal materials in industrial applications, where the increased catalyst longevity and overall energetic efficiency allowed by photothermal systems could potentially offset the costs of specialty plasmonic catalysts. This could prove particularly useful for reactions that already use a precious metal as their catalyst, where changes in morphology and reaction mediation could allow the process to be run photothermally.

While previous examples use metallic species which have strong plasmonic capabilities, a 2023 report from the Zhang group designed a new photothermal nanoparticle (Figure 6c).^47^ They wanted to improve the photothermal properties of Co species, which typically suffer from low absorption coefficients, limiting potential applicability. However, improvements in light absorption were made by encapsulating the Co nanoparticles with organic ligands. Using this novel photothermal agent, they were able to perform the photooxidation of benzyl alcohol with 7.8-fold reaction efficiency compared to purely thermal conditions. This method is complementary to Jiang’s approach to the oxidation of benzylic alcohols, as it preferentially affords the ester over the aldehyde.

## 3 Couplings, Rearrangements, and Diels–Alder Reactions

Hydrogenation and oxidation reactions represent industrially relevant transformations that often rely on catalyst incorporation alongside high temperatures. Using photothermal conversion agents increased the energy and reaction efficiency while simplifying conditions and increasing selectivity. Additionally, many transformations in medicinal chemistry and academia could benefit from incorporating a photothermal agent. Indeed, transformations such as the Claisen rearrangement and Diels–Alder reaction, which often proceed at elevated temperatures, have been successfully facilitated by photothermal conversion.

**Figure 7:**
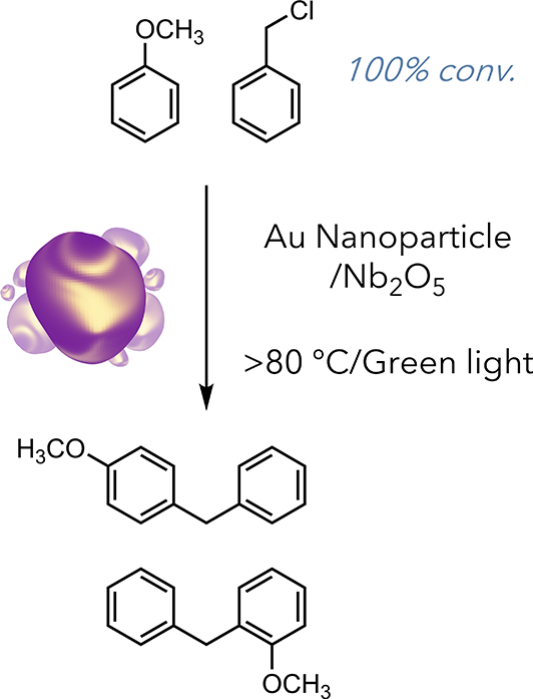
Friedel–Crafts alkylation of anisole by benzyl chloride.

The Scaiano laboratory is a pioneer in this area, demonstrating both dual photothermal and catalytic approaches and solely photothermal methods for achieving organic transformations.^48,49^ In 2017, they used novel gold and niobium-based nanocomposites as Lewis acid catalysts to facilitate the Friedel–Crafts alkylation of anisole (Figure 7).^49^ This reaction, typically carried out at elevated temperatures (>150 °C), proceeded readily under their conditions, exhibiting a considerably lower bulk temperature of 80 °C.

**Figure 8:**
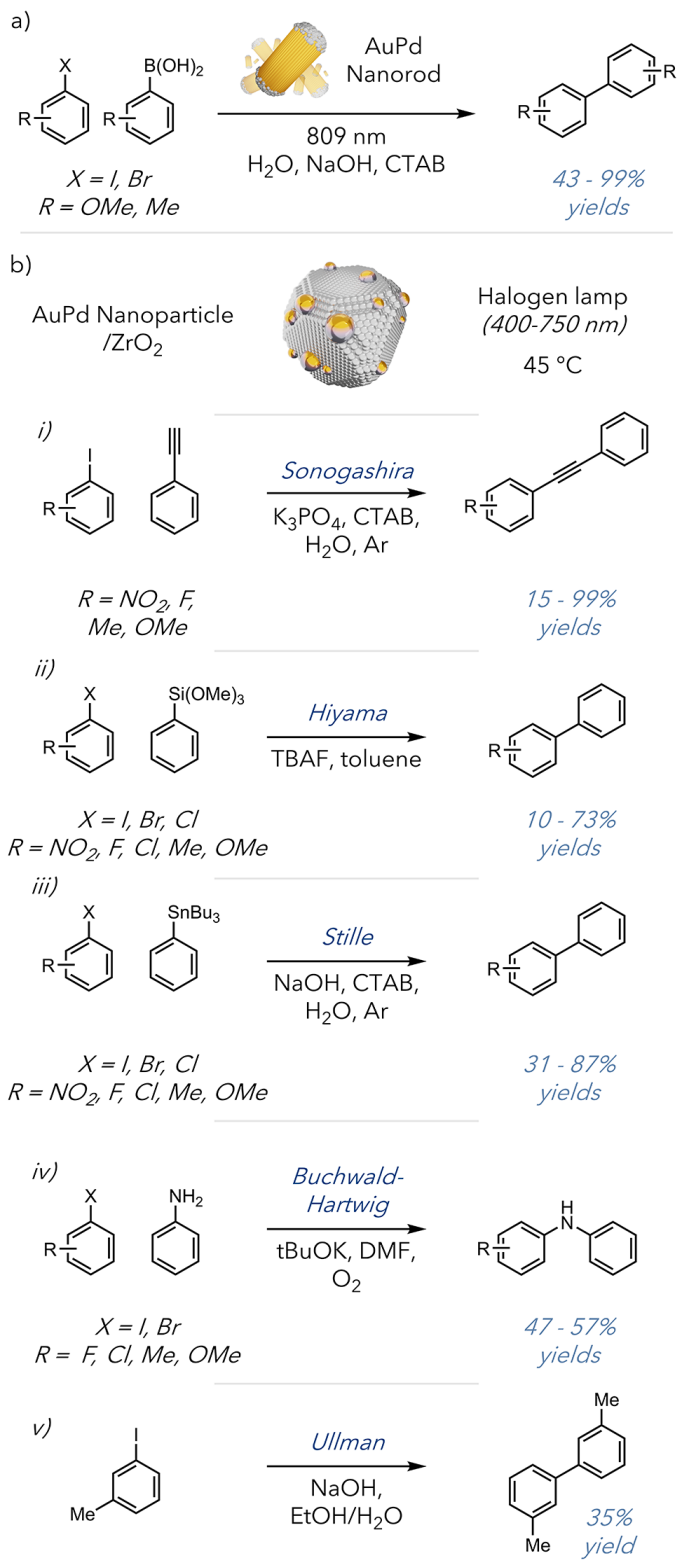
a) Suzuki coupling. b) AuPd nanoparticle-assisted (i) Sonogashira coupling, (ii) Hiyama coupling, (iii) Stille coupling, (iv) Buchwald–Hartwig coupling, and (v) Ullman coupling.

Another significant report from the Yan laboratory in 2013 describes using bimetallic AuPd nanostructures to perform Suzuki coupling reactions (Figure 8a).^50^ They could use high-intensity laser light and focused sunlight to achieve their transformations with similar overall yields. Following this initial report, there has been significant interest in plasmonically mediated cross-coupling reactions, with a wide array of plasmonic metallic species and irradiation sources able to successfully facilitate the transformation.^51−53^ The general applicability of these conditions was later illustrated in a report that used AuPd nanoparticles to promote several different classes of cross-coupling reactions—Sonogashira, Stille, Hiyama, Ullman, and Buchwald–Hartwig (Figure 8b).^54^ They found that in each instance, except for the Sonogashira coupling, conversion was significantly higher when the AuPd nanoparticles were used than when Pd nanoparticles alone were used. In the Ullman and Buchwald–Hartwig couplings examples, yields were almost doubled (35% vs 16% and 50% vs 35%, respectively), demonstrating a definitive enhancement in reaction efficiency. While application of these species does not supplant the use of precious metals, increased reaction efficiencies and the lower effective loadings that nanoparticles afford allow for the enhancement of an industrially relevant application. This demonstrates that while photothermal conversion may be used to access entirely new reactivity, it can additionally be used in well-studied schemes to produce enhancements. Additionally, this protocol offers a highly efficient and milder approach to some of the most synthetically valuable reactions used in organic chemistry.

**Figure 9:**
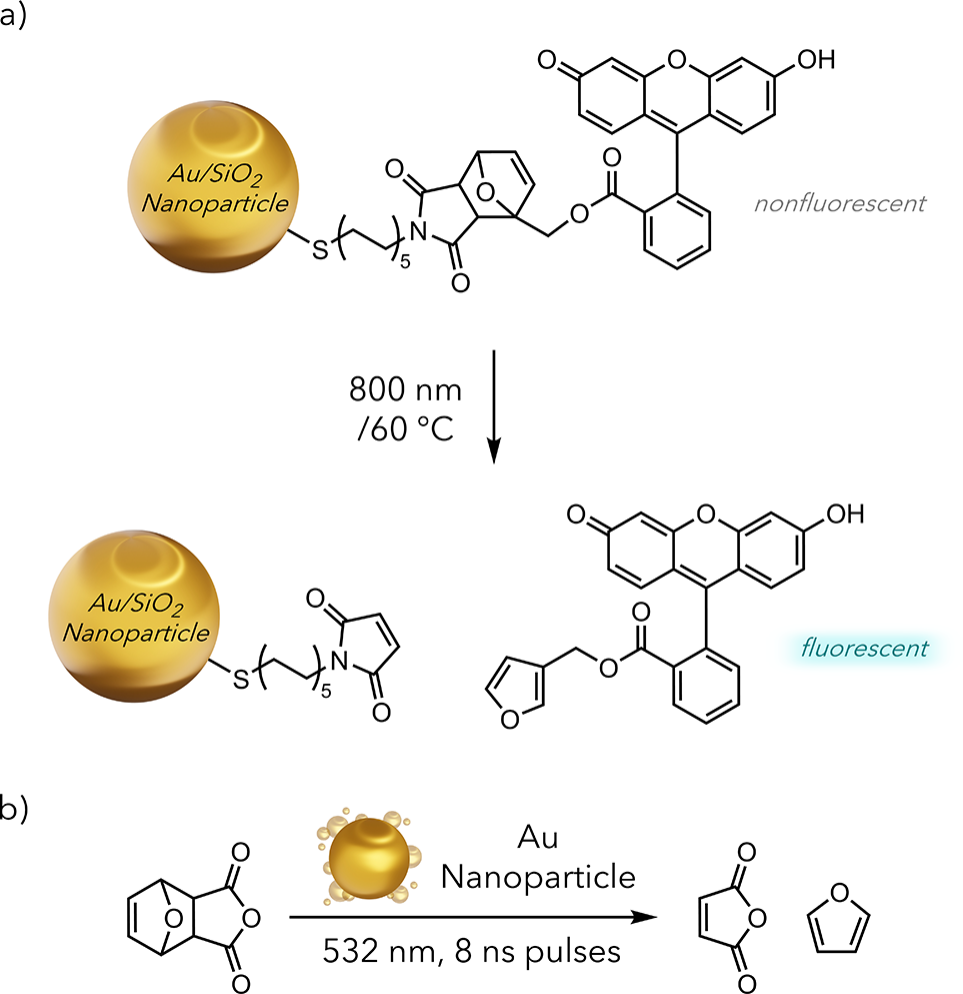
a) Reverse Diels–Alder covalently bound to a gold nanoparticle. b) Reverse Diels–Alder mediated by a gold nanoparticle.

Numerous transformations require high temperatures and proceed without a catalyst, such as the Diels–Alder and Claisen rearrangements. Such reactions seem uniquely suited to photothermal promotion, with the first report appearing in 2009.^55^ The Branda group devised a photothermally induced retro-Diels–Alder reaction to develop a photocontrolled drug release system (Figure 9a). Their method involved covalent linkages between the gold nanoparticle and the furan/maleic anhydride-derived Diels–Alder adduct to release a fluorescein unit upon irradiation. Therefore, they monitored the reaction progress using the fluorescence emission intensity. Under thermal conditions, this reaction proceeds at ∼60 °C to 25% conversion after 30 min. In contrast, the photothermally mediated reaction achieved complete conversion in the same time.

A photothermal system could offer tremendous advantages regarding drug delivery systems, wherein effective dosage and targeted delivery are long-term challenges. One potential challenge is that, for biomedical systems, low-intensity near-infrared light is often used on account of its high penetration depth. However, many plasmonic and carbonaceous materials do not absorb or absorb poorly at these wavelengths, precluding their use. In contrast, molecular photothermal agents, such as heptamethylene blue or indocyanine green, have been used successfully at these wavelengths as photothermal therapeautics. New syntheses incorporating them into a drug release scaffold would need to be developed, however. An alternative approach would be to provide modifications to plasmonic or carbonaceous species to shift their absorption profile. Additionally, many reports use species covalently attached to the nanoparticle. However, the Lear group demonstrated that these reactions could proceed readily when kept as discrete entities in relatively dilute solutions (Figure 9b).^56^ This proved significant because of the highly sequestered nature of photothermal heating.

**Figure 10:**
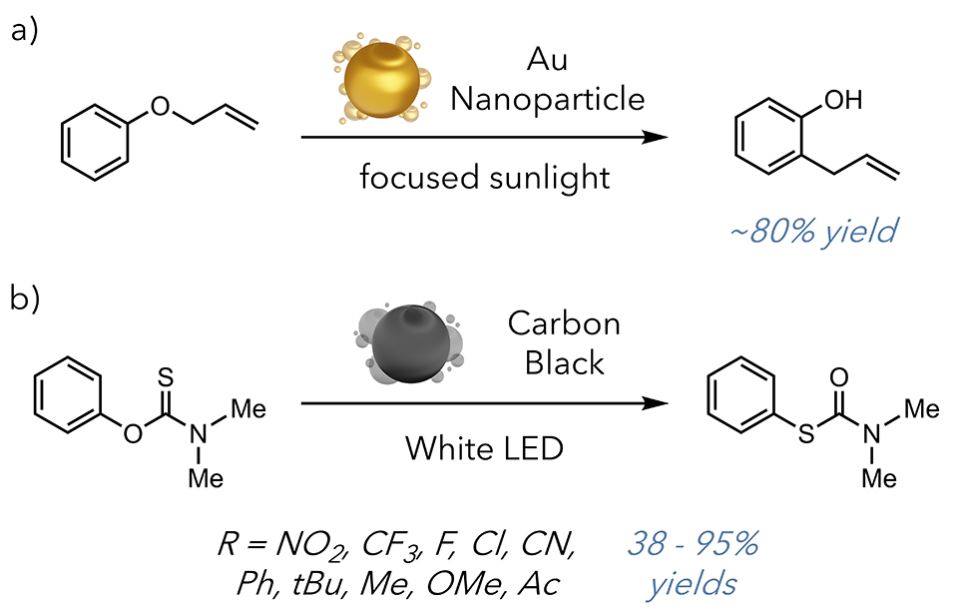
a) Claisen rearrangement. b) Newman Kwart rearrangement.

Appreciable distances between a substrate and nanoparticle allow nonproductive heat to be released into the system, causing a potential drop in reaction efficiency. This presents a potential challenge for organic reactions which are typically run dilute or require certain low-boiling solvents to work, as contact with the nanoparticle and effective heat transfer may become difficult. However, the Pillai group demonstrated that photothermal agents could function in a system without direct interaction between nanoparticle and substrate (Figure 10a).^57^ Their 2023 report used a specialty reactor that isolated plasmonic gold nanoparticles from allyl phenyl ether to exempt any competitive plasmonic effects in their system. Using this reactor and focused sunlight, they achieved a highly efficient Claisen rearrangement. They obtained an 80% yield of their rearranged product after 2 hours of exposure to focused sunlight. This method produced a 2-fold rate enhancement compared to traditional methods, which involve heating the reaction to 250 °C. This work demonstrates how photothermal agents can perform challenging reactions using sustainable energy sources. This work is additionally unique, as it essentially uses plasmonic species as a source to generate bulk heat, not relying on the close interaction between nanoparticle and substrate but instead using the nanoparticles as a way to generate a high bulk temperature. While this may seem counterintuitive to some of the advantages inherent to photothermal approaches, it demonstrates how it may also be leveraged to perform green chemistry. They were able to use sunlight to promote these reactions, presenting a possible way for photothermal agents to be used as a green way to establish high-temperature processes using biorenewable energy.

While most work in the photothermal conversion/catalysis space has focused on metallic nanoparticles, the Stache laboratory demonstrated a photothermally promoted Newman-Kwart rearrangement using carbon black as a photothermal agent (Figure 10b).^58^ Carbon black is a low-cost and easily accessible industrial byproduct. Unlike metallic nanoparticles with strict LSPR frequencies, carbonaceous materials absorb broad-spectrum irradiation to induce photothermal conversion. The Newman Kwart rearrangement is an intramolecular O to S migration well known for its high activation energy barriers (35–43 kcal/mol) and sensitivity to substitution patterns. Stache and co-workers demonstrated that incorporating small amounts of carbon black and exposure to broad-spectrum white LED light for short irradiation periods enabled high yields. They also showcased changes in product selectivity based on wavelength-specific irradiation, highlighting the tunability of the strategy. These reports are indicative of the vast potential that photothermal reaction mediation possesses.

## 4 Polymers

**Figure 11:**
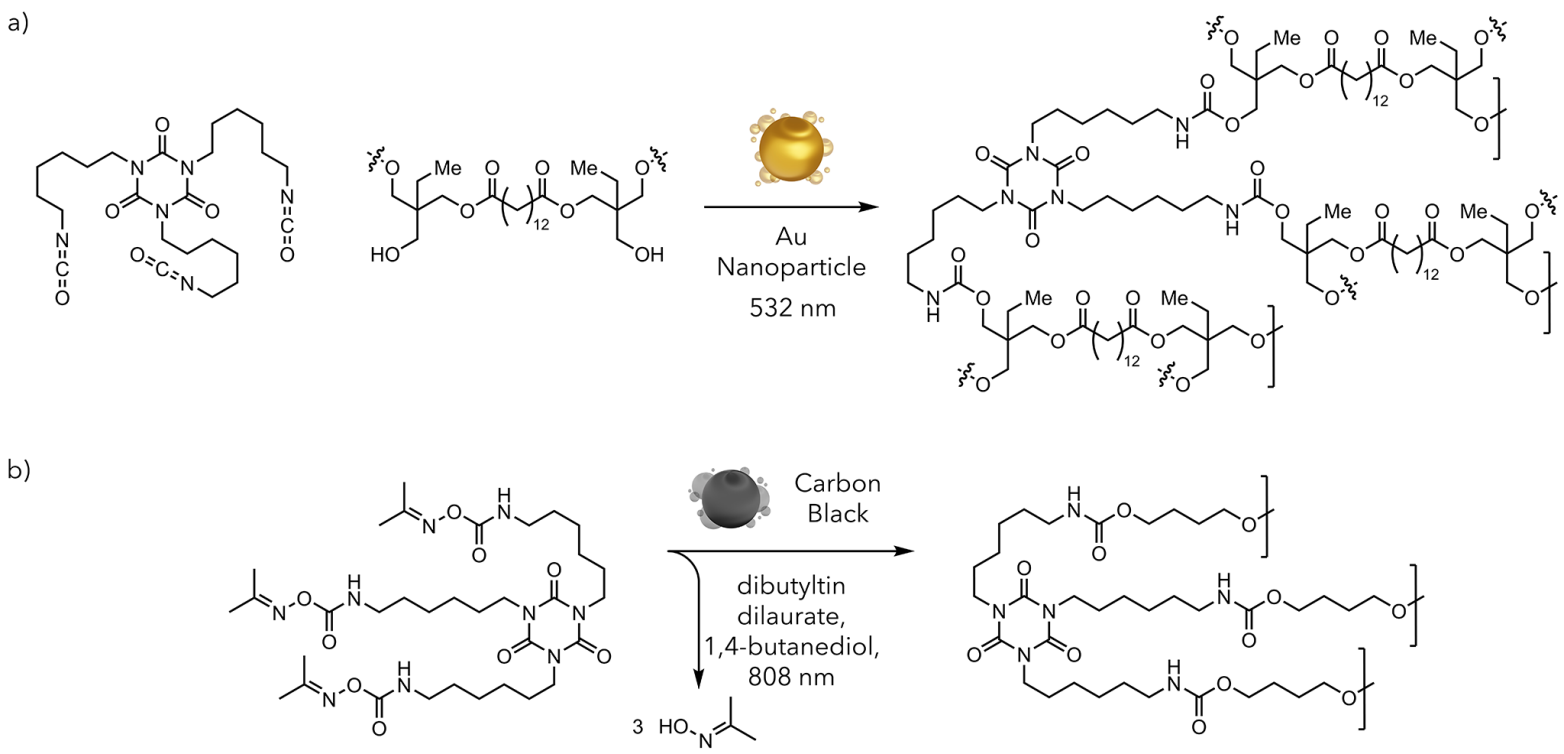
a) Polyurethane polymerization of alcohols and isocyanates. b) Polyurethane polymerization of blocked isocyanates.

Photothermal conversion combines the advantages of photocatalysis (spatiotemporal control, tunability by intensity and wavelength of light, etc.) with the ubiquity of thermally promoted processes. Polymer synthesis, processing, and recycling are highly relevant to everyday life and reliant on thermal mediation. This thermal dependence has resulted in diverse photothermal approaches to polymer chemistry, including nanolithography, shape-memory polymer synthesis, and circular recycling methodologies. The Zaleski group demonstrated the potential for photothermally mediated polymerization in a 2011 report focusing on the surface modification of gold nanoparticles.^59^ While the paper focused on nanoparticle development, they found that photothermal heating could be leveraged to induce free radical polymerization of their modifying enediyne species. The Lear group, previously cited for their contributions to photothermal Diels–Alder chemistry, has significantly contributed to this area. They induced polymerization between alcohols and isocyanates to form polyurethanes, creating a highly efficient curing protocol (Figure 11).^60,74^ While they anticipated that high local temperatures would readily promote curing, they were surprised to note a “billion-fold” rate enhancement of the reaction compared to traditional heating. After performing a series of calculations, they determined that the surface of the nanoparticle was likely reaching 600–800 K (325–525 °C) while the bulk temperature remained much lower.

**Figure 12:**
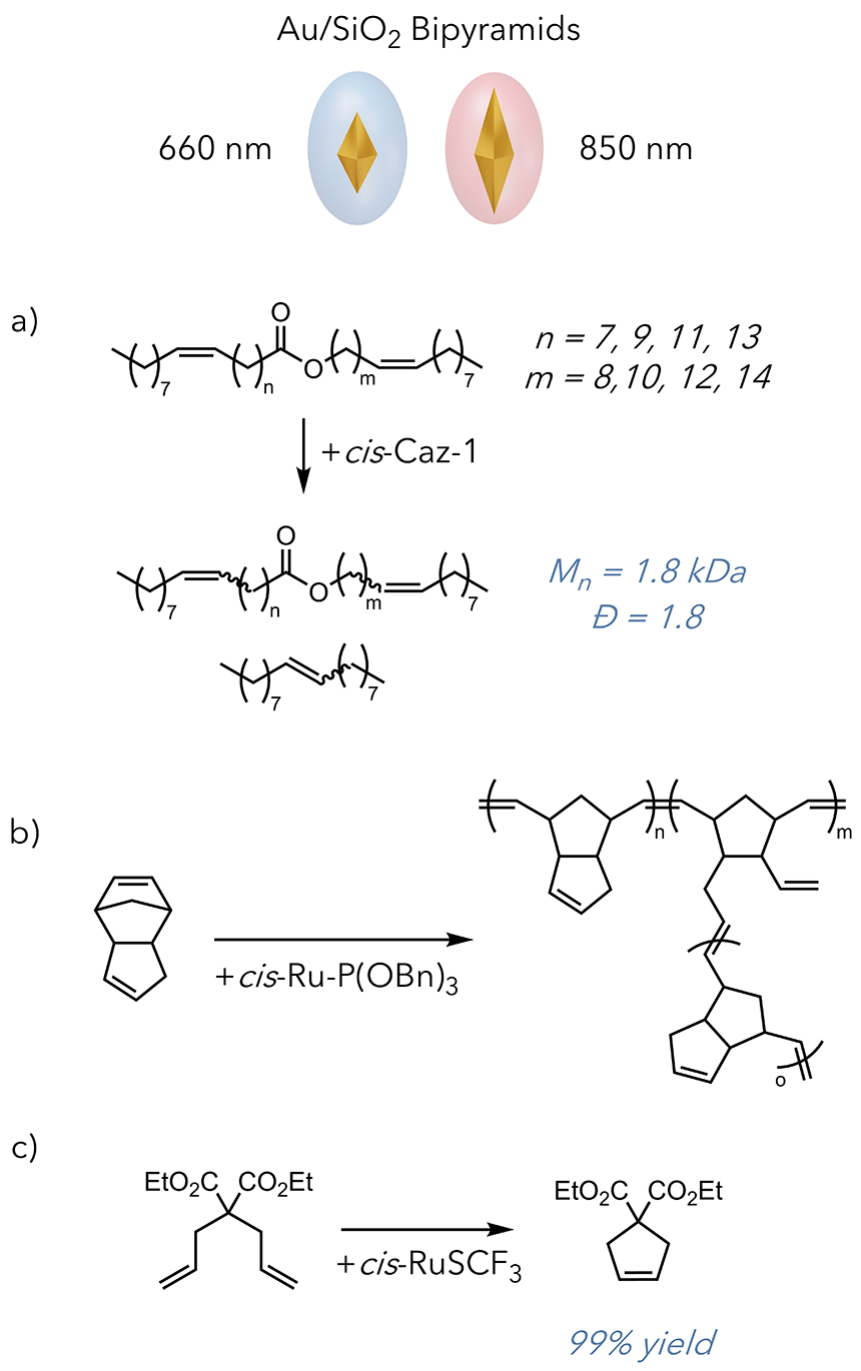
Use of two types of silica-capped gold bipyramids to enable (i) acyclic diene metathesis (ADMET) of jojoba oil, (ii) ROMP of DCPD, and (iii) ring-closing metathesis (RCM) of diethyl diallyl malonate.

**Figure 13:**
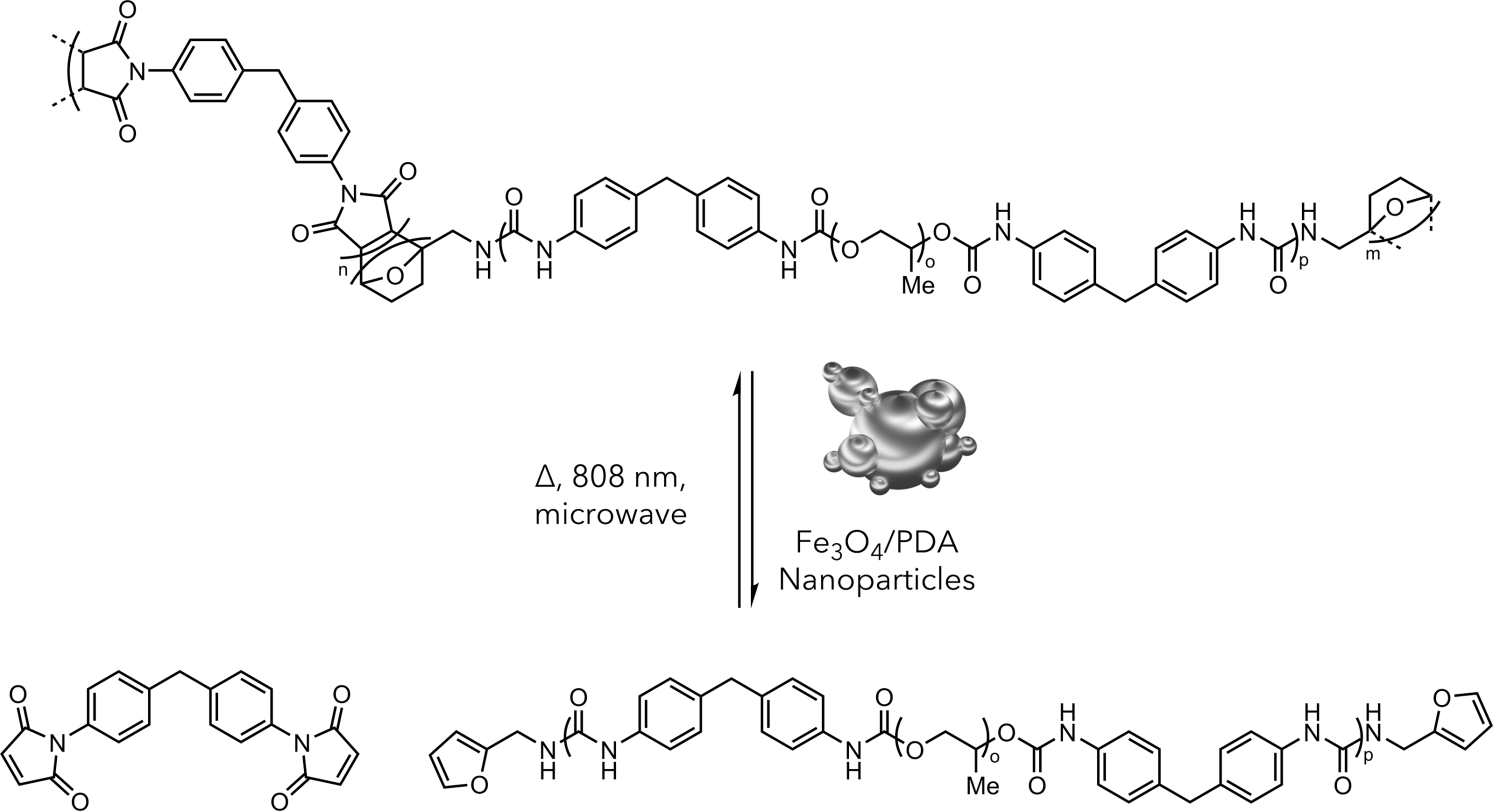
Self-healing polyurethane by copolymerization with Diels–Alder moieties and incorporation of Fe_3_O_4_/PDA nanoparticles.

Several groups have also facilitated photothermally promoted ring-opening metathesis polymerizations (ROMPs). While the recovery of heterogeneous material is possible in organic reactions, increasing viscosity during polymerization often eliminates this possibility in macromolecular processes. Therefore, any plastics made using these methods contain metal contaminants. The ROMP of dicyclopentadiene has been photothermally mediated successfully by gold nanoparticles, which the Lemcoff and Weizmann groups reported in 2023, where they additionally demonstrated the acyclic diene metathesis polymerization (ADMET) of jojoba oil (Figure 12).^61^ This report also examined the latent photoresponsive properties inherent to materials synthesized with added photothermal agents, as selective irradiation to light allows for postpolymerization modifications to occur. This idea has been manifested in several engineering applications, which examine how photothermal agent incorporation may allow for shape-memory or “smart” polymers and the ability to perform 3-D printing and lithography using light.^62−64^ Latent photoresponsive properties in polymers containing photothermal materials presents immense potential for dynamic materials whose synthesis and material properties can be controlled via irradiation. A recent example from the Feng group used magnetite nanoparticles embedded within polyurethane alongside Diels–Alder units to induce photoresponsive self-healing events (Figure 13).^64^ Notably, they found that the incorporation of the nanoparticle led to improved mechanical properties and excellent healing capability. Additionally, the incorporation of photothermally active polydopamine units enabled the use of NIR light, greatly increasing the applicability of this protocol.

**Figure 14:**
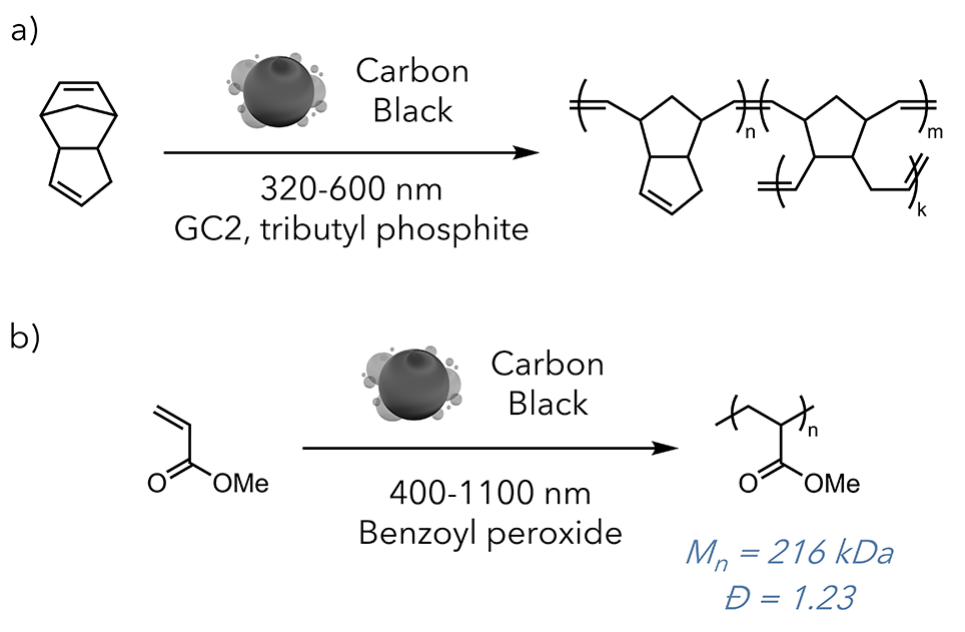
a) Frontal ring-opening metathesis polymerization (FROMP) of dicyclopentadiene (DCPD). b) Radical polymerization of methyl acrylate.

The Sottos group used carbon black to mediate the frontal polymerization of dicyclopentadiene (Figure 14a).^65^ Not only could they use a small photothermal agent loading (1 wt %) but they also observed a 30-fold decrease in energy required to achieve this transformation compared to traditional methods. The Esser-Kahn laboratory similarly used carbon black to initiate the free radical polymerization of acrylates and methacrylates (Figure 14b).^66^ Interestingly, they observed a distinct effect on the microstructure of the polymer. By performing thermogravimetric analysis and taking SEM images, they found that the photothermal polymers had a highly organized morphology at the microscale. This organization resulted in an ∼5 °C increase in the glass-transition temperature and demonstrated the unique effect photothermal heating has, even on uncontrolled polymerizations. This, along with work by Stache, Sottos, and Lear, indicates that inexpensive and accessible carbon black can act as a highly effective photothermal agent instead of more costly specialty materials.

**Figure 15:**
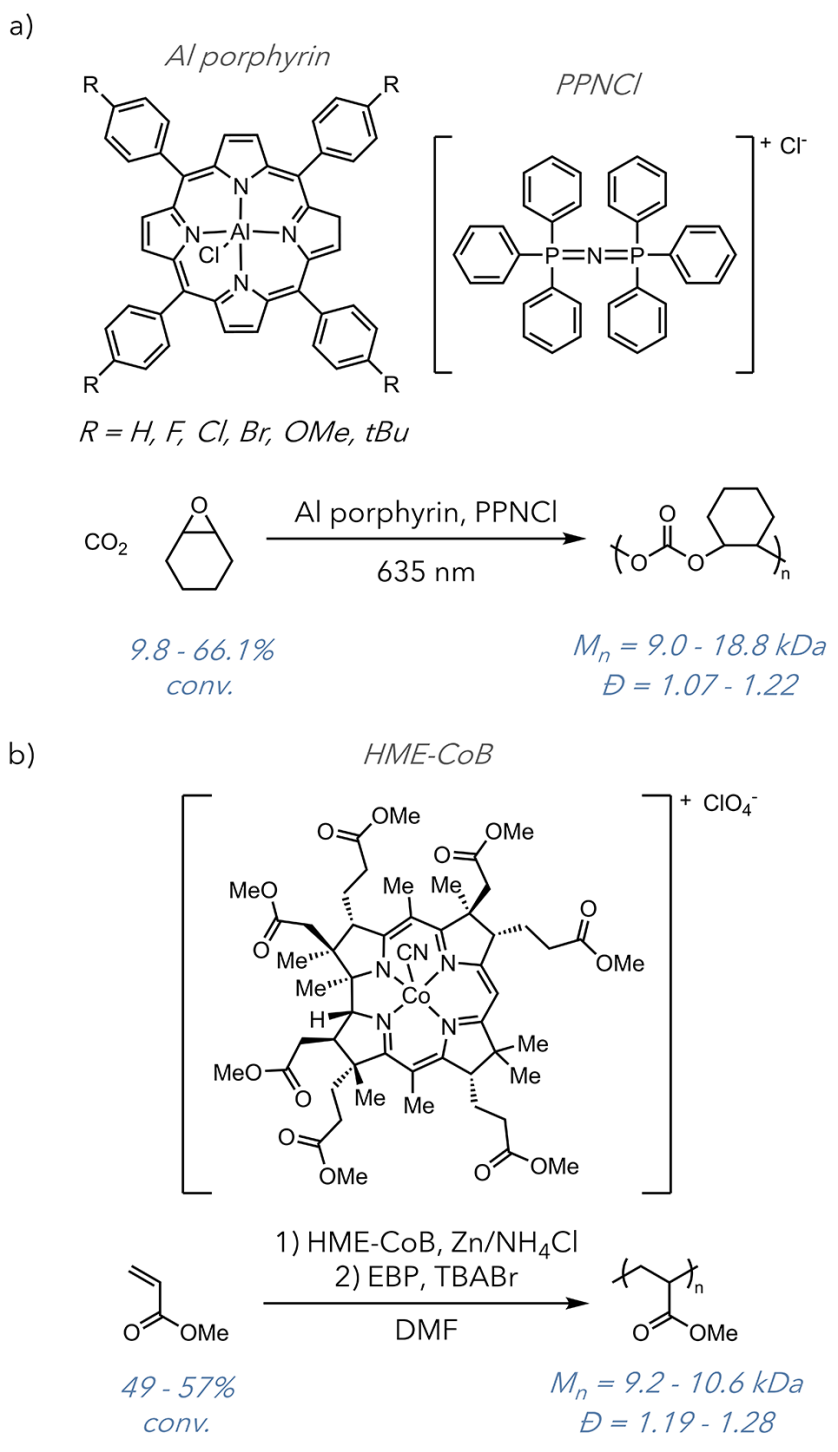
a) Ring-opening copolymerization of carbon dioxide and cyclohexene oxide by an Al porphyrin catalyst. b) Photothermally controlled ATRP of methyl acrylate.

While still an emergent approach, two reports have leveraged metallomacrocycles for dual catalytic and photothermal polymerization. In 2022, the Wang group used an aluminum-centered porphyrinic macrocycle to promote the photothermal ring-opening copolymerization of carbon dioxide and epoxides (Figure 15a).^67^ They used red laser irradiation to produce a bulk temperature increase in the range of 65–90 °C. They found that the photothermal capacity of their catalyst aided in reaction efficiency, increasing catalyst turnover by 2-fold. Because the degree of intramolecular motion profoundly affects photothermal conversion, they introduced additional phenyl rotors to the porphyrin to increase molecular flexibility.

A new report from Stache and co-workers details the use of the vitamin B_12_-derived cobyrinate macrocycle, which displays dual photothermal and catalytic properties (Figure 15b).^68^ This is the first photothermally promoted controlled radical polymerization report. As a photocontrolled process, they achieved a high degree of temporal control over the polymerization without sacrificing polymerization control compared to traditional atom-transfer radical polymerization (ATRP) systems. They could also use much lower intensity light with simple LED strips. Conjugated organic macrocycles are commonly used as photothermal agents in therapeutic settings, with species such as indocyanine green and heptamethylene blue frequently seeing use. The similar structural motifs in these complexes, also present in many catalyst scaffolds such as porphyrins and cobyrinates, indicate an untapped potential among many catalytic species. These reports indicate exciting new possibilities for polymer synthesis and offer long-term improvements for spatiotemporal control, increased catalyst efficiency, and monomer scope.

## 5 Conclusions and Outlook

It is clear from the existing literature that photothermal conversion can function in a highly diverse manner and promote remarkable reactivity. A lingering question remains regarding plasmonic materials—to what degree are specific reactions being promoted plasmonically versus photothermally? While this inquiry has been addressed in a few reports and several detailed here via clever experimental design, many variables in the system still call for elucidation. Simplifications may arise in an eventual shift to predominately nonplasmonic materials, which have demonstrated immense potential in the limited examples presented here. Carbonaceous materials offer several schematic advantages over their metallic counterparts, removing the need for specialty synthesis or fears over eventual deformation. The Lear group has released an excellent report detailing how another alternative could be a material such as magnetite, which is similarly low-cost and unaffected by heat.^69^ Furthermore, several groups have developed methods which aim to address several of the challenges associated with metallic nanoparticles. Synthesizing multiple shapes and sizes of plasmonic species enables the use of a broader wavelength range and broad-spectrum light. Challenges associated with nanoparticle deformation and degradation upon heating have been addressed via the use of protective coatings or embedding in a macromolecular scaffold.

**Figure 16:**
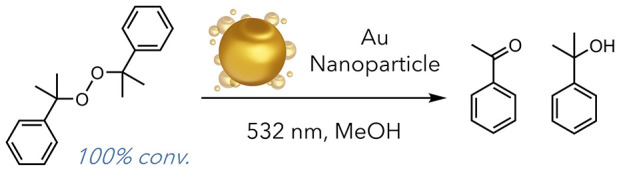
Decomposition of dicumyl peroxide by gold nanoparticles.

Certain limitations still exist, such as expensive or specialty materials, a need for high-intensity light, and an uncertainty in the surface temperatures of these materials. Efforts have been made within the community to address these issues successfully. While nanoscale thermometry is an ongoing area of research, its applicability to photothermal systems remains limited.^70,71^ Particle surface temperatures have instead been estimated using well-established reactions and qualitative calculations. In 2011, the Scaiano laboratory was able to probe the surface temperature of gold nanospheres by monitoring the decomposition of dicumyl peroxide (Figure 16).^48^ It was used as a model reaction due to its known reactivity and activation barrier for cleavage (34.4 kcal/mol). They used pulsed laser light and gold nanospheres to decompose dicumyl peroxide with 100% conversion in less than 2 min. Using kinetic data, they could extract an estimated surface temperature of ∼500 °C, a massive temperature increase generated solely through light exposure. These results indicated that photothermal nanoparticles could generate large thermal gradients to drive reactivity. These incredible surface temperatures calculated by Lear and Scaiano indicate the potential for the photothermal facilitation of tremendously challenging reactions.^60^

Additionally, distinguishing between photothermal conversion and hot carrier processes in plasmonic systems increases complexity and makes studying these materials as photothermal agents more difficult. Several reports have emerged which aim to deconvolute these processes to make their relative contributions more clear. A 2020 report used varying laser intensities and beam diameters to try to determine the relative contributions of each process.^43^ The report aimed to outline a series of relatively simple procedures which could be used to gauge relative pathways but ultimately concluded that there remain multiple challenges for quantitatively determining each aspect. A more recent report, published in 2024, investigated plasmonic heating in electrochemical settings, monitoring plasmonic electrodes.^42^ Cyclic voltammetry and potentiometry enabled them to investigate how irradiation affects the charge distribution in the system. This allowed them to determine relative heating Vs. charge carrier generation. An inherent challenge in these systems is their nanoscale profile and extremely short excitation lifetimes. Furthermore, interactions between nanoparticles may result in differing relaxation pathways, making studies of single nanoparticles, which are often performed due to simplicity, nonrepresentative of the actual system. This proves challenging not only for nanoscale thermometry but also for elucidating which relaxation path may prevail under a given set of conditions. In order to enhance the general understanding and provide insight into how photothermal reactivity may be better controlled, new analytical techniques and experiments should be developed to elucidate global versus local temperature effects and which energetic decay pathways are operating under a given set of conditions. This could lead to new scientific advancements in the field and make the system more approachable to researchers using photothermal conversion.

Ultimately, the extent to which photothermal agents can promote challenging reactivity is still under exploration. The extraordinary effect of nanoscale heating is continuously documented in all of these examples. Much like the unique reactivity observed when a reaction is run in a microdroplet, heating on the nanoscale is not well studied or understood enough to adequately hypothesize what unique effects it may produce.^72,73^ Additionally, several thermodynamically feasible and operationally simple transformations have remained out of reach due to prohibitively high activation energy barriers. While theoretically possible, reactions have been abandoned because of prohibitive side reactivity or poor yields. More progress within the field may eventually lead to addressing these issues.Characterization of the specific energy profiles generated at the surface of these species as well as their differing methods for energy transfer should help address some of these challenges and lead to expansion and exploration within the field. While indeed a field in its infancy, photothermal conversion for organic synthesis shows incredible promise.
